# Ultrasound Guided Parasternal Block for Perioperative Analgesia in Cardiac Surgery: A Prospective Study

**DOI:** 10.3390/jcm12052060

**Published:** 2023-03-06

**Authors:** Giuseppe Pascarella, Fabio Costa, Giulia Nonnis, Alessandro Strumia, Domenico Sarubbi, Lorenzo Schiavoni, Annalaura Di Pumpo, Lara Mortini, Stefania Grande, Andrea Attanasio, Giovanni Gadotti, Alessandro De Cassai, Alessia Mattei, Antonio Nenna, Massimo Chello, Rita Cataldo, Felice Eugenio Agrò, Massimiliano Carassiti

**Affiliations:** 1 Unit of Anaesthesia and Intensive Care, Fondazione Policlinico Universitario Campus Bio-Medico, 00128 Rome, Italy; 2 Unit of Anaesthesia and Intensive Care, Ospedale dei Castelli, Ariccia, 00040 Rome, Italy; 3 Unit of Anaesthesia and Intensive Care, Ospedale Sant Orsola, 40138 Bologna, Italy; 4 Unit of Anaesthesia and Intensive Care, Azienda Ospedaliera San Camillo Forlanini, 00152 Rome, Italy; 5 Unit of Anaesthesia and Intensive Care, University Hospital of Padua, 35128 Padua, Italy; 6 Unit of Cardiac Surgery, Fondazione Policlinico Universitario Campus Bio-Medico, 00128 Rome, Italy

**Keywords:** parasternal block, regional anaesthesia, cardiac surgery, cardiac anaesthesia, pain management

## Abstract

Ultrasound guided parasternal block is a regional anaesthesia technique targeting the anterior branches of intercostal nerves, which supply the anterior thoracic wall. The aim of this prospective study is to assess the efficacy of parasternal block to manage postoperative analgesia and reduce opioid consumption in patients undergoing cardiac surgery throughout sternotomy. A total of 126 consecutive patients were allocated to two different groups, receiving (Parasternal group) or not (Control group) preoperative ultrasound guided bilateral parasternal block with 20 mL of 0.5% ropivacaine per side. The following data were recorded: postoperative pain expressed by a 0–10 numeric rating scale (NRS), intraoperative fentanyl consumption, postoperative morphine consumption, time to extubation and perioperative pulmonary performance at incentive spirometry. Postoperative NRS was not significantly different between Parasternal and Control groups with a median (IQR) of 2 (0–4.5) vs. 3 (0–6) upon awakening (*p* = 0.07); 0 (0–3) vs. 2 (0–4) at 6 h (*p* = 0.46); 0 (0–2) vs. 0 (0–2) at 12 h (*p* = 0.57). Postoperative morphine consumption was similar among groups. However, intraoperative fentanyl consumption was significantly lower in the Parasternal group [406.3 ± 81.6 mcg vs. 864.3 ± 154.4, (*p* < 0.001)]. Parasternal group showed shorter times to extubation [(191 ± 58 min vs. 305 ± 72 min, (*p*)] and better performance at incentive spirometer with a median (IQR) of 2 raised balls (1–2) vs. 1 (1–2) after awakening (*p* = 0.04). Ultrasound guided parasternal block provided an optimal perioperative analgesia with a significant reduction in intraoperative opioid consumption, time to extubation and a better postoperative performance at spirometry when compared to the Control group.

## 1. Introduction

Due to the improvement in life expectancy, heart surgery has become increasingly common among elderly population. However, due to the frailty and comorbidities of these patients, perioperative complications are frequent. Moreover, the impact of anaesthetics and analgesic drugs play an important role on the perioperative course [1]. 

Post-operative pain is mainly related to median surgical sternotomy; it is intense and difficult to control, especially in the immediate few hours after surgery [2]. It requires high doses of opiates that may lead to different side effects, such as nausea, vomiting, respiratory issues and postoperative delirium. Furthermore, high anaesthetic drug administration can cause a delay in extubating time and weaning from mechanical ventilation. Moreover, sympathetic stimulus and chest pain may induce an important reduction in thoracic excursion, finally facilitating pulmonary infections [3]. 

Recently, many thoracic fascial plane blocks have been proven to be a valid option in controlling pain in cardiac, thoracic and breast surgery [4,5,6,7]. 

The sternal region is innervated by the anterior branches of intercostal nerves, which arise from the anterior branches of the spinal nerves from T1 to T11 [8]. Parasternal block is a recent block which targets these branches due to the injection of local anaesthetic between the pectoral major and the intercostal muscles, proximal to the sternum. To obtain a good analgesic cover, the block needs to be performed bilaterally with a spread from the II to the VI intercostal spaces. 

Several studies have investigated the parasternal block in cardiac patients trying to understand its impact on intra- and post- operative pain [9,10,11,12,13]; however, most of the studies are retrospective or based on a small number of patients.

The aim of this prospective study is to assess the efficacy of parasternal block to manage postoperative analgesia, reduce opioid consumption and improve respiratory function in patients undergoing cardiac surgery.

## 2. Materials and Methods

This study was approved by Campus BioMedico Hospital Ethical Committee (protocol number 20/20 PAR ComEt CBM) and was registered on clinicaltrias.gov (NCT04319588).

A total of 126 patients who underwent elective cardiac surgery were enrolled between November 2019 and March 2020. Written informed consent was obtained from all the subjects. Inclusion criteria to participate included: patients aged 18 years or older, ASA physical status I-IV and candidates for elective cardiac surgery with median sternotomy. Exclusion criteria included: allergy to local anaesthetics, site of puncture infection, weight < 30 Kg, impaired cerebral function and patient’s disapproval.

All patients received general anaesthesia conducted with sevoflurane 2%, fentanyl 3–5 mcg/kg, remifentanil continuous infusion 0.1 mcg/kg/min (depending on clinical judgment of the anaesthetist), rocuronium 0.8–1 mg/kg and propofol 2% continuous infusion (in patients who underwent extracorporeal circulation). The administration of adjunctive boluses of fentanyl (1 mcg/kg) intraoperatively was decided by the anaesthetists based on the hemodynamic parameter variations suggesting pain, such as a rapid increase in blood pressure and heart rate in the absence of fluid loading or amine infusion.

Patients were freely allocated in two groups:

- The “*Parasternal*” group received ultrasound guided bilateral parasternal block after general anaesthesia induction and an infiltration with local anaesthetic of the surgical drainage sites at the end of surgery.
- The “*Control*” group received just the infiltration with local anaesthetic of the surgical drainage sites at the end of surgery.

Drainages sites’ infiltration was provided since the parasternal block does not target the sub-phrenic area where the drainages exit the skin. Moreover, we performed it in both groups to eliminate potential bias generated by patients’ upper abdominal pain perception. 

Both groups received the same protocol of multimodal perioperative analgesia, which included: dexamethasone 0.1 mg/kg i.v. intraoperative; ketorolac 60 mg/24 h i.v. and paracetamol 1 g every 8 h i.v., postoperatively. In addition, morphine 2 mg i.v. was administered in the case of postoperative NRS pain scores ≥ 6.

A spirometry evaluation was performed both immediately before surgery and in the postoperative period with the aim to evaluate respiratory function after the surgical intervention and to compare to baseline values. This evaluation was performed with the TriFlo Inspiratory Exerciser^®^, a device which consist of three air columns with different weights and coloured balls that move up when the patient inhales. 

At the end of the operation, all patients were transferred to the intensive care unit (ICU) for weaning from invasive mechanical ventilation and immediate postoperative monitoring. 

Data collected: anthropometric data; chronic opiate medications; diabetes mellitus; pre-existing pulmonary conditions; type of surgery—aortocoronary bypass, off pump aortocoronary bypass, valvular surgery and ascending aortic surgery; length of surgery; postoperative pain at extubation and after 6–12–24 h on a 0–10 numeric rating scale (NRS) expressed as maximum pain experienced during that period of time; postoperative opiates consumption: time to first morphine bolus administration (from the patient awakening); total morphine consumption during the first 24 h after surgery; intraoperative opiate consumption (fentanyl, remifentanil); time to extubation; side effects—nausea and vomiting; delirium (using the Mini Mental State Examination); and respiratory performance with the TriFlo expressed as the number of balls moved up before and after the surgery. All the data in the ICU (e.g., NRS, postoperative consumption, pulmonary function, etc.) were collected by the ICU nurses specialized in the management of cardiac surgery patients.

### 2.1. Block Execution

**Figure 1:**
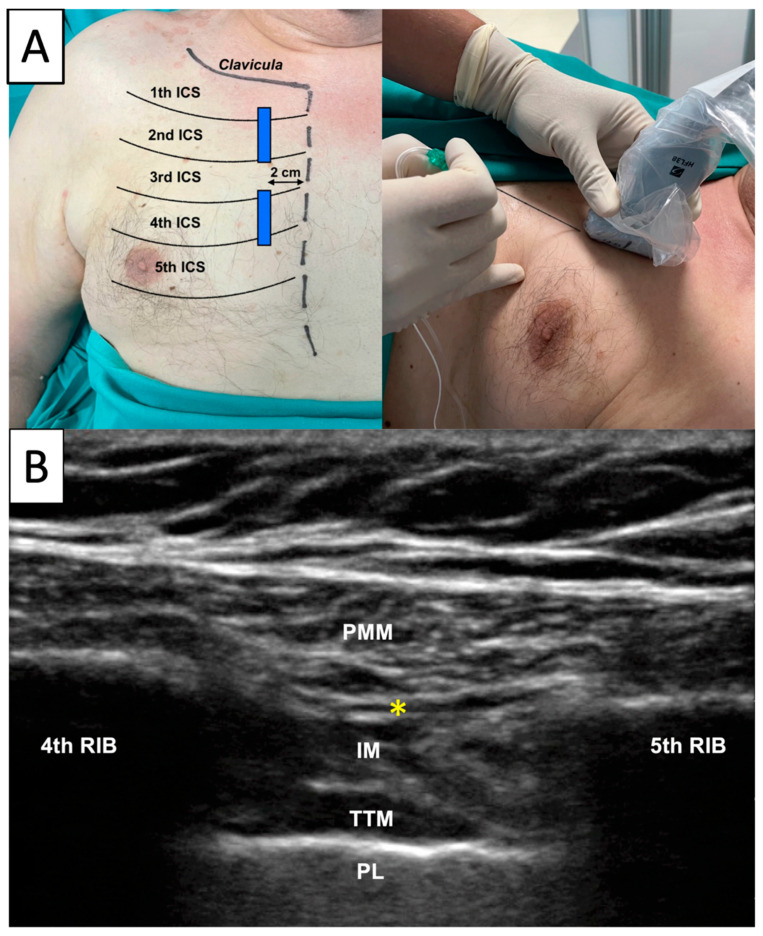
Parasternal Block Execution. *Legend:* A high frequency ultrasound probe was positioned immediately lateral to the sternum identifying the second and the fourth intercostal spaces (**A**). Then, an echogenic 100 mm needle (Stimuplex ultra-360, BBraun Deutschland GmbH & Co., Melsugen, Germany) was advanced through the skin with an in-plane approach and an injection of 10 mL 0.5% ropivacaine was performed between pectoral major and intercostal muscles for every intercostal space, bilaterally, with a total dose of 200 mg of ropivacaine (**B**). PMM: pectoral major muscle; IM: intercostal muscles; TTM: transversus thoracis muscle; PL: pleura; *: point of injection.

The patients in the Parasternal group received bilateral ultrasound guided parasternal block immediately after induction of general anaesthesia and intubation [14]. The block was executed with aseptic technique right after patient intubation and before surgical incision. A high-frequency ultrasound probe was positioned immediately lateral to the sternum, identifying the second and the fourth intercostal spaces (Figure 1A). Then, an echogenic 100 mm needle (Stimuplex ultra 360, BBraun Deutschland GmbH & Co., Melsugen, Germany) was advanced through the skin with an in-plane approach and an injection of 10 mL 0.5% ropivacaine was performed between pectoral major and intercostal muscles, bilaterally, with a total dose of 200 mg of ropivacaine (Figure 1B). Success of the block was confirmed by the presence of the double hypoechogenic V sign indicative of correct presence of local anaesthetic between the two muscular fasciae [15]. All the blocks were performed by two expert anaesthesiologists in regional anaesthesia who had already performed more than 50 parasternal blocks before enrolling the first patient. 

At the end of the surgery, both groups received a local infiltration of the surgical drainages with ropivacaine 0.25% 20 mL performed by the surgeon.

### 2.2. Statistical Analysis

To calculate a sample size, we focused on our primary hypothesis that perioperative analgesia is improved with the parasternal block. We estimated the density of pain scores (mean 2; SD 1.5) based on published data regarding the use of parasternal block for cardiac surgery [16]. To simulate power, we used the truncated Gaussian distribution with range 0–10; standard deviation = 1.5; PARASTERNAL group mean = 2. Under these assumptions and 2-sided α = 5%, we simulated 10,000 trials with sample size of 63 per group. With an overall sample size of 126 subjects, we have at least 90% power to detect group differences in pain as small as approximately 1. 

Statistical analysis and graphical presentation were obtained thanks to the use of the GraphPad Prism 8 software (GraphPad Software Inc., San Diego, CA, USA).

The values of continuous quantitative variables are expressed as mean ± standard deviation (SD); the values of discrete variables are expressed as median and interquartile range (IQR). Qualitative variables are expressed as number of observations and percentage of distribution.

The parametric distribution of numerical variables was evaluated using the Shapiro–Wilk normality test. Difference between groups was assessed by T-Student test for continuous parametric variables, while Wilcoxon–Mann–Whitney U test was used when appropriate. Bonferroni–Dunn correction has been applied to multiple repeated measures in order to reduce the risk of type 1 error.

Categorical variables were compared with Pearson’s χ^2^ test. The level of statistical significance was set for *p* value < 0.05. 

## 3. Results

A total of 126 patients were enrolled in this study. In the Parasternal group, the median age was 67 ± 10 years, while in the Control group was 70 ± 10; both groups had a higher prevalence of males, and the mean body mass index (BMI) was 26.5 kg/m^2^ ± 2.1 in the Parasternal group and 27.8 kg/m^2^ ± 1.1 in the Control group.

Aortocoronary bypass in extracorporeal circulation (CEC) was the most performed intervention overall (57% in Parasternal group and 65% in Control group), followed by valvular surgery (30% and 25%, respectively); less frequent were off-pump aortocoronary bypasses, combined bypasses plus valvular surgery and thoracic aorta aneurysm. Surgical average duration was 230.7 ± 53.5 min in the Parasternal group, and 213 ± 40.8 min in the Control group (Table 1).

Intraoperative fentanyl consumption emerged to be significantly higher in the Control group with a mean of 864.3 mcg compared to 406.3 mcg in the Parasternal group (*p*-value < 0.001). Intraoperative remifentanil administration was not statistically significative between the two groups.

Median (IQR) postoperative pain at awakening, expressed as maximum NRS scale value (range 0–10), was 2 (0–4.5) in the Parasternal and 3 (0–6) in the Control group (*p* = 0.07); in the next 6 h it was 0 (0–3) in the Parasternal and 2 (0–4) in the Control group, while it was 0 in both groups during the following hours. However, pain was always under 4 at 48 h after extubation (mild pain) in both groups. 

Postoperative opiates were requested by 30% of patients in the Parasternal group and by 29% of patients in the Control group. Furthermore, both groups showed no difference in the median (IQR) consumption of postoperative morphine during the first 24 h (0 (0–2) mg). Furthermore, the median (IQR) time to first opioid administration was similar (30 (10–45) mg for parasternal group vs. 30 (11–60) mg for control group) (Table 2).

Mean extubation time from admission in ICU was 191 ± 48 min in the Parasternal group and 305 ± 62 min in the Control group (Table 1).

Respiratory performance was evaluated by the TriFlo Inspiratory Exerciser and was expressed in the number of balls moved up during inspiration one hour after extubation. The median number of balls moved up was 2 (1–2) in the Parasternal group versus 1 (1–2) in the Control group. 

Side effects, such as nausea, vomiting and delirium, were present in a small percentage of patients (2–3%) in both groups. Data regarding respiratory performance and side effects are reassumed in Table 3.

## 4. Discussion

This study was conducted on an extremely heterogeneous population, including every patient who underwent cardiac surgery; we tried to confront two different groups with different analgesic strategies in terms of pain management, recovery capacity and postoperative outcome.

Due to the increased life expectancy, heart surgery has become frequent in the elderly population; in our study, while the mean age of our patients was 70 years old, we faced many patients aged 80 years or more and were affected by many comorbidities. To best manage this frail population, it is mandatory to devise an anaesthetic plan aimed at minimizing the impact on the patient’s vital functions and homeostasis [17]. Perioperative strategies must be adopted to reduce surgical invasiveness, post-operative pain, anaesthetic drugs usage and promote early respiratory recovery and patient-autonomous mobilization [1]. 

Pain, in fact, plays a central role in this therapeutic process; it reduces thoracic excursion and influences respiratory functional recovery, finally promoting pulmonary complications, such as pneumoniae, pleural effusion and lung atelectasis. 

Different regional anaesthetic protocols have been proposed to better control sternal pain after cardiac surgery. Starting from neuraxial techniques, different approaches have been developed to target the thoracic fascial plane where intercostal nerves from T1 to T11 lay, such as the pectoral blocks [18,19], the serratus anterior plane block [20], the transversus thoracic muscle block [21], the erector spinae block [22] and the parasternal block. All these blocks contribute to obtaining a better control of thoracic pain in heart surgery, reducing recovery time and improving dismission time [23]. 

The parasternal block is one of the more promising fascial blocks to manage sternal pain as described in the recent studies conducted by Sepolvere et al. [24,25]. 

**Figure 2:**
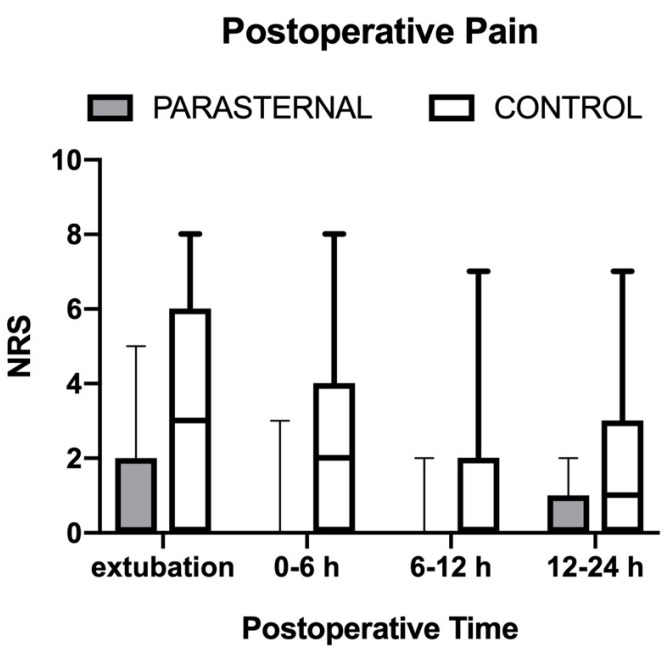
**Postoperative Pain.** *Legend:* Maximum postoperative (numeric rating scale) pain scores in both study groups reported during four postoperative intervals. Values are median (horizontal bars), IQR (box) and range (whiskers). Denotes statistical significance (*p* < 0.05).

In our study, post-operative pain, expressed with an NRS scale from 0 to 10, was very low in both groups in absence of a significant statistical difference (Figure 2). These results are similar to the study by Lee et al. [11], as they found a marginal decrease in postoperative pain levels, although they use a longer acting local anaesthetic (liposomal bupivacaine).

Additionally, postoperative opiate consumption was low in both groups without any significant statistical difference, with a mean morphine consumption of 1 mg overall. This result could be correlated to the long and complex type of surgery and the timing of the block execution. In fact, we observed a mean surgical time between 3 and 4 h; moreover, a phase of recovery from anaesthesia and weaning from mechanical ventilation in ICU before weaking up was always needed. Therefore, extubation often happened several hours after the critical phase of acute postoperative pain, which is particularly intense in the immediate period after the end of the surgery, as shown in the study of Zubrzycki et al. [2]. Moreover, considering the timing of the block execution, patients were often extubated several hours after the execution of the parasternal block when the analgesic effect is not at the apex anymore, which is in accordance with the studies demonstrating that the analgesic effect of the block usually last between 5 and 12 h [26,27]. Nevertheless, the small difference in pain detected at patients awakening could have had a role in the prolonged extubation time observed in the Control group. Performing the block during the postoperatively probably impacted more on pain, but some difficulties may rise, such as the oedema of the nearby tissues and the sterile medication applied on the wound itself, which both may be complicating factors for ultrasound visualization. Moreover, and most important, performing the block before the surgery has an important impact on diminishing intraoperative opiates consumption, as demonstrated by Bloc et al. [28]. 

Another interesting result regards intraoperative fentanyl consumption: intraoperative administration in the Control group was twice the dose used in the Parasternal group. This result is in line with a recent study conducted in 2020 [16], which compared two groups of cardiac surgery patients who underwent parasternal block before and after the intervention. Authors found a significant reduction in intraoperative opioid administration in the preoperative Parasternal group, suggesting an important pre-emptive and intraoperative effect of the block. Limiting the intraoperative dosage of opiates could lead to rapid weaning from mechanical ventilation, early extubation and lucid weaking up, which improves patient outcomes. 

Moreover, considering the low NRS scores and the successful postoperative pain control in both groups, the analgesic intraoperative regimen adopted probably had an important influence on the postoperative period. Multimodal pre-emptive analgesia with opiates, FANS, regional anaesthesia, corticosteroids, and paracetamol plays a fundamental role in preventing the development of surgical pain [29]. 

Notably, time to extubation could be affected by several other factors, such as preoperative conditions, i.e., pre-existing pulmonary and neurologic disorders; surgical factors, such as intraoperative and postoperative bleeding; and postoperative conditions, such as arrhythmia or bleeding. Nevertheless, we did not register significant differences in patients’ characteristics and perioperative conditions that could have affected or delayed time to extubation. 

It must be said that other parameters, such as intraoperative heart rate and pressure values, use of vasopressor drugs, conscious state at awakening and respiratory complications, have not been evaluated in our study.

Pulmonary performance is another interesting result to underline. Postoperative chest pain is usually evaluated with the patient in a supine position in the absence of movement. Respiratory evaluation could be a way to evaluate parasternal block efficacy when the thorax is moving. 

Every patient underwent the TriFlo Inspiratory Exerciser test immediately after extubation and weaning from mechanical ventilation, which was compared to the baseline test performed immediately before surgery. Data showed that patients in the parasternal block moved up a mean of one ball more than the Control group (2 and 1 balls, respectively). This result may suggest a better analgesic effect of the parasternal block during thoracic excursions and consequently a better respiratory performance in the postoperative period; this implies better blood oxygenation and oxygen saturation, better airway clearance and a lower risk of postoperative pneumonia. Moreover, earlier extubation and less mechanical ventilation time could also have improved respiratory performance in the Parasternal group.

This study has several limitations. Firstly, it is difficult to show a clinically meaningful result for postoperative pain, which was one of our primary outcomes, when baseline postoperative pain scores are already low. However, these results confirm those of Lee et al. [11].

Secondly, we did not record pain on movement, although performance at spirometer may be considered an inversely proportional indirect index of chest pain.

Moreover, we did not investigate the correlation between perioperative analgesia and other outcomes, such as, days of ICU stay, total hospitalization time and incidence of chronic postoperative pain. At the same time, we did not correlate time to extubation and postoperative spirometer performance with the incidence of respiratory complications, leading, for example, to supplemental oxygen therapy or mechanical ventilation. We expect several future studies focus in depth on these aspects.

Another limit of the study is broad inclusion and exclusion criteria without reporting more baseline characteristics that could have influenced the primary outcome, such as pre-existing chronic pain and neuropathy, although the incidence of chronic opioid medications was similar among groups. Nevertheless, some important pre-existing conditions have been investigated and discussed.

Lastly, postoperative multimodal analgesia included on-demand administration of i.v. morphine, although recommendations indicate the oral route is preferable for opioid intake. However, this is not always possible as patients undergoing cardiothoracic surgery may have dysphagia and an increased aspiration risk in the first postoperative period [30]. 

In this regard, a solution could be represented by the administration of sublingual sufentanil through a patient-controlled analgesia (PCA) system, which has already proved to be effective and safe in thoracic surgery [31].

## 5. Conclusions

Ultrasound guided parasternal block seems to be an effective, safe and easy to perform technique in patients undergoing cardiac surgery under sternotomy. Although it did not significantly affect postoperative analgesia, it showed a relevant reduction in intraoperative opioid consumption and a better postoperative performance at spirometry compared to the Control group. Further studies are expected to confirm these findings and explore the medium- and long-term impact of this technique on postoperative morbidity.

## Figures and Tables

**Table 1:** Patients’ characteristics.

	Parasternal (63)	Control (63)	*p*-Value
Age (years)	67 ± 10	69.6 ± 10	0.17
Sex (M/F)	33/30	35/28	0.8
BMI (kg/m^2^)	26.7 ± 4	26.5 ± 3.3	0.9
Chronic opiates medication	2 (3%)	3 (5%)	>0.9
Diabetes Mellitus	13 (21%)	12(19%)	>0.9
Pre-existing pulmonary disorders	11 (17%)	10 (16%)	0.88
Type of surgery			
CABG	36 (57%)	41 (65%)	0.6
CABG off pump	3 (5%)	4 (6%)	
Valvular surgery	19 (30%)	16 (25%)	
CABG + valvular surgery	4 (6%)	1 (2%)	
Thoracic aorta aneurysm	1 (2%)	1 (2%)	
Surgery duration (min)	230.7 ± 53.5	213 ± 40.8	0.08

Values are expressed in mean ± standard deviation or in number of patients (%); CABG = coronary artery bypass surgery.

**Table 2:** Main Outcomes.

	Parasternal	Control	*p*-Value
**Intraoperative fentanyl (γ)**	406.3 ± 81.6	864.3 ± 154.4	**<0.001**
**Intraoperative remifentanil (γ)**	336.1 ± 13.1	338.3 ± 13.5	0.3367
**Postoperative pain** (NRS max 0–10)			
Extubation	2 (0–4.5)	3 (0–6)	0.07
0–6 h	0 (0–3)	2 (0–4)	0.46
6–12 h	0 (0–2)	0 (0–2)	0.57
12–24 h	1 (0–2)	2 (0–3)	0.69
**Postoperative opiates consumption**			
Yes	19 (30%)	18 (29%)	0.8
No	44 (70%)	45 (71%)	
**Time to first opioid (min)**	30 (10–45)	30 (11–60)	0.6
**Morphine consumption 0–24 h (mg)**	0 (0–2)	0 (0–2)	>0.9

Values are expressed in mean ± standard deviation; median (interquartile range); number of patients (%); NRS (numeric rating scale).

**Table 3:** Secondary Outcomes.

	Parasternal	Control	*p*-Value
**Time to extubation (min)**	191 ± 48	305 ± 62	**<0.001**
**Pulmonary performance (balls moved up)**			
Basal	3 (2–3)	3 (2–3)	0.9
After extubation	2 (1–2)	1 (1–2)	**0.045**
**Side effects**			
Nausea	2 (3%)	1 (2%)	–
Vomit	1 (2%)	2 (3%)	–
Delirium	1 (2%)	2 (3%)	–

Values are expressed in mean ± standard deviation; median (interquartile range); number of patients (%).

## Data Availability

The data presented in this study are available on request from the corresponding author due to privacy and ethical reasons.
